# Graphene Dot–ZnO Hybrid Nanostructures as High-Performance Chemiresistive Sensors for H_2_S Detection

**DOI:** 10.1021/acsomega.5c13473

**Published:** 2026-04-10

**Authors:** Federica Bucolo, Daniela Iannazzo, Nesrine Hafiene, Consuelo Celesti, Roberto Di Pietro, Ulderico Wanderlingh, Sebastiano Vasi, Giovanni Neri

**Affiliations:** † Department of Engineering, 18980 University of Messina, Messina 98166, Italy; ‡ Department MIFT, University of Messina, Messina 98166, Italy; § OPENFIS S.R.L.-Spin off accademico, University of Messina, Laboratorio A2AT3, Viale F. Stagno D’Alcontres 31, Messina 98166, Italy

## Abstract

The development of sensitive, selective, and low-cost gas sensors for hazardous pollutants remains a crucial challenge in environmental monitoring. Among gaseous pollutants, hydrogen sulfide (H_2_S) has been shown to negatively influence ecosystem dynamics. The novelty of this study lies in the synergistic integration of graphene dots (GDs) with ZnO, which significantly enhances the sensing performance while enabling efficient operation at a reduced temperature. The synthesized materials were comprehensively characterized using X-ray diffraction (XRD), scanning electron microscopy (SEM), Fourier-transform infrared (FTIR) spectroscopy, UV–visible spectroscopy, Raman spectroscopy, photoluminescence spectroscopy (PL), dynamic light scattering (DLS), and thermogravimetric analysis, confirming the successful formation of the GD–ZnO hybrid structure. The fabricated thick-film sensors, tested in the 0.125–4 ppm of H_2_S concentration range, exhibited an optimal operating temperature of 200 °C and a linear and fast response with a good sensitivity and recovery time. Compared to pure ZnO, the GD–ZnO composite in a 1:1 ratio not only demonstrated higher sensitivity and selectivity toward H_2_S but also achieved efficient gas detection at a significantly lower operating temperature. The GD–ZnO (1:1) composition demonstrated the best performance, showing excellent repeatability, stability, and discrimination against interfering gases, such as CO, SO_2_, NO_2_, and H_2_. Overall, the enhanced sensing performance, characterized by a sensitivity of 2.8582 × 10^–3^, low operating temperature, and reliable response–recovery behavior, highlights the strong potential of the GD–ZnO nanocomposite for practical implementation in compact, low-power, and cost-effective H_2_S monitoring devices for environmental and industrial safety applications.

## Graphical abstract

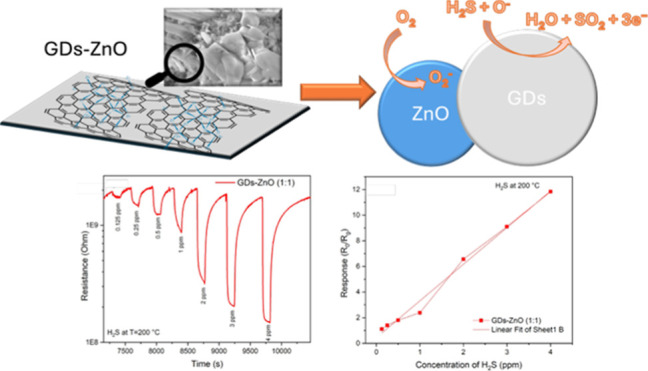

## 1 Introduction

One of the major challenges faced by modern society are the detection, control, and reduction of hazardous contaminants released into the atmosphere. Continuous monitoring of air pollutants is crucial to mitigate climate change and its negative effects on human health and on environment and also to preserve cultural heritage materials that are highly vulnerable to chemical degradation.^1^ Common air pollutants include carbon dioxide (CO_2_), carbon monoxide (CO), hydrogen sulfide (H_2_S), ozone (O_3_), ammonia (NH_3_), nitrogen and sulfur oxides (NO_*x*_ and SO_*x*_), particulate matter (PM_2.5_ and PM_10_), and volatile organic compounds (VOCs). Among them, sulfur-based compounds such as H_2_S are of particular concern because of their toxicity, corrosive nature, and strong environmental impact.^2^ Although H_2_S is naturally produced by anaerobic bacterial activity in oxygen-depleted ecosystems such as swamps, as well as by geothermal phenomena including volcanoes and hot springs, industrial and human sources, such as petroleum refining, natural gas processing, paper production, and various chemical manufacturing processes, represent the primary contributors to atmospheric emissions.^3^

According to occupational safety guidelines, the threshold limit value (TLV) for H_2_S is in the low-ppm range (a round 5–10 ppm for short-term exposure), highlighting the need for sensitive and reliable detection at subppm concentrations.^4^

Given its high toxicity and the potential for material degradation even at low concentrations, real-time detection of H_2_S has become increasingly important. Metal oxide semiconductor (MOS) sensors are the most widely used H_2_S detectors because of their low cost, ease of fabrication, high sensitivity, and reliable stability.^5^ In addition, MOS sensors offer robust performance under harsh environmental conditions and are easily integrated into compact and low-power sensing platforms, making them particularly suitable for continuous and on-site monitoring applications.^6^ However, their main limitations include poor selectivity and a requirement for relatively high operating temperatures. In contrast, conducting-polymer-based sensors offer operation at lower temperatures but often suffer from low sensitivity, limited selectivity, and limited operational lifetime. Recent studies on polyaniline (PANI)-based chemiresistive sensors have highlighted significant improvements using flexible architectures, tailored nanostructures, and polymer–nanomaterial composites; nevertheless, their performance remains strongly influenced by environmental factors and long-term stability issues.^6,7^

To overcome these limitations, carbon-based nanomaterials have emerged as attractive alternatives for gas sensing applications due to their exceptional physical, chemical, and electronic properties, combined with a high surface-to-volume ratio and tunable surface chemistry.^8^ Among them, graphene-derived materials such as graphene oxide (GO), reduced graphene oxide (rGO), graphene quantum dots (GQDs), and carbon nanotubes (CNTs) have shown great promise in the development of high-performance gas sensors.^8,9^ Their surfaces can be easily functionalized to enhance interaction with target molecules, either through covalent bonding to oxygen-containing groups introduced by oxidative treatments or via noncovalent π–π stacking with aromatic structures.^10^

Recent studies have also demonstrated the effectiveness of graphene-based composites for the detection of volatile organic compounds (VOCs), where the integration of graphene with metal oxide nanoparticles has significantly improved the selectivity and sensitivity of sensors, opening new avenues for environmental monitoring. For example, GO/ZnO nanocomposites exhibited remarkable selectivity and sensitivity for VOC detection, making them ideal for environmental monitoring. Similarly, GQDs were demonstrated to have enhanced sensing properties for VOCs, highlighting their superior performance compared to bulk graphene. These advancements illustrate the potential of graphene-based materials in multifunctional gas sensing applications, capable of detecting both VOCs and hazardous gases like H_2_S.^11,12^

Graphene-based H_2_S sensors can generally be categorized into three main types: graphene/metal, graphene/metal oxide, and graphene/polymer hybrid systems.^13^ Concerning metal sensors, various metals have been widely investigated for gas sensing applications, including studies supported by Density functional theory (DFT) calculations. Metal-decorated semiconducting metal oxides with different nanostructures (e.g., nanoparticles, nanowires, nanorods, nanosheets, nanoflowers, and microspheres) have been employed in the development of high-performance gas sensors characterized by high sensitivity, fast response and recovery times, low operating temperatures, and ultralow detection limits.^14,15^ The incorporation of metal oxides or polymers into graphene-based frameworks often leads to synergistic effects, improving sensitivity, selectivity, and stability while lowering the operating temperature and response/recovery times.^16^ These nanomaterials, owing to their unique features, are fragments of monolayers or a few layers of graphene sheets with lateral size generally below 20 nm.^17^ Due to their very small dimension, graphene-based nanostructures have emerged as one of the most promising platforms for next-generation gas sensors, particularly for the selective and low-level detection of toxic gases such as H_2_S.^18^

To further improve the electrical conductivity of graphene-based materials, zinc oxide nanoparticles (ZnO NPs) have been vertically grown on chemically converted graphene films, yielding sensors capable of detecting 2 ppm of H_2_S in oxygen even at room temperature.^19^ Recent studies have shown that modifications to ZnO can significantly enhance its sensing performance. For example, ZnO nanoclusters on fluorine-doped tin oxide (FTO) electrodes exhibited remarkable linear responses to very low H_2_S concentrations (62.5–1000 ppb) with stable performance, though at an elevated operating temperature (∼330 °C).^20^ Additionally, hierarchical ZnO structures such as flower-like or mesoporous morphologies have demonstrated faster response times and higher sensitivity owing to increased surface area and more exposed reactive sites.^21^ In particular, graphene dots (GDs), the next generation of graphene-based nanomaterials, were selected due to their unique 0D structure, high density of edge-active sites, and tunable surface functional groups, which collectively enhance their interaction with electron-withdrawing gas molecules such as H_2_S.^22^ Their discrete band gap and efficient charge-transfer capabilities enable a stronger modulation of the sensing layer’s electronic properties compared to graphene oxide or bulk graphene.^23^ Furthermore, when integrated with ZnO, these nanomaterials facilitate the formation of effective heterojunctions that amplify depletion-layer variations, improving the sensitivity and selectivity while maintaining stable operation. These attributes make GDs a compelling choice for advanced chemi resistive H_2_S sensing applications.^24^

The excellent electrocatalytic activity, availability, low cost, and environmental compatibility of semiconducting ZnO NPs^25^ make them ideal candidates for integration with GDs, which offer superior electronic properties and additional active sites for gas interaction. This choice is further supported by the findings of Lee et al., who demonstrated that GDs with discrete band gaps significantly enhance gas–surface interactions and charge-transfer processes, enabling ultralow NO_2_ detection limits down to the ppb level and markedly improved selectivity, thereby confirming the strong sensing enhancement achievable through GD–metal oxide heterostructures.^26^

In this context, this work focuses on the development and characterization of GD–ZnO nanocomposites with GD-to-ZnO weight ratios as hybrid sensing materials for H_2_S detection. By integrating the semiconducting properties of ZnO with the high electron mobility and surface reactivity of GDs (Figure 1), the resulting composites aim to achieve improved sensitivity, selectivity, and lower operating temperatures compared to conventional ZnO-based sensors.^27^

**Figure 1:**
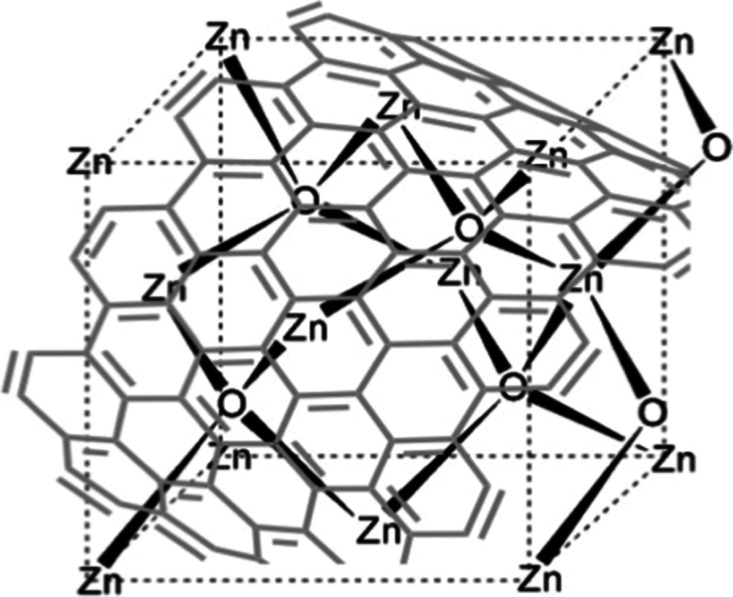
Schematic representation of the GD–ZnO nanocomposite.

## 2 Experimental Section

### 2.1 Materials

Zinc oxide nanopowder (ZnO, analytical grade) was purchased from Sigma-Aldrich (St. Louis, MO, USA) and used without further purification. GDs have been synthesized from multiwalled carbon nanotubes (MWCNTs) following a previously reported procedure.^28^ All aqueous solutions were prepared using deionized water (resistivity ≥18.2 MΩ·cm).

### 2.2 Synthesis of Graphene Dots (GDs)

GDs were synthesized according to the previously reported procedure.^28^ Briefly, pristine MWCNTs have been subjected to acidic exfoliation using a solution of HNO_3_/H_2_SO_4_ in a 1:3 ratio. The resulting suspension was placed in an ultrasonic bath at 60 °C for 4 days. After dilution, the mixture was filtered using a 0.1 μm Millipore membrane under vacuum. Then, after a treatment with a sodium hydroxide (NaOH) solution until neutral pH and centrifugation, the mixture was purified using a dialysis bag with a molecular weight (MW) of 12,000 Da. The final GD dispersion exhibited strong blue photoluminescence under ultraviolet (UV) irradiation (λ = 365 nm) and was stored at 4 °C until further use.

### 2.3 Synthesis of GD–ZnO Nanocomposites

GD–ZnO hybrid nanocomposites were prepared via a solution-phase approach. A ZnO NP dispersion (6 mg) obtained by ultrasonication of commercial ZnO nanopowder in deionized water for 30 min allowed the achievement of a homogeneous colloidal suspension. Subsequently, the ZnO dispersion (0.6 mg/mL in the ratio 1:1) was added dropwise to the GD solution (10 mL, 0.6 mg/mL) under magnetic stirring and maintained under continuous agitation for 1 h to ensure uniform mixing and interfacial interaction between ZnO NPs and GDs. The mixture was then heated at 180 °C for 12 h under reflux conditions to promote the anchoring of ZnO onto the GD surface and to enhance composite formation. After being cooled to room temperature, the suspension was concentrated under reduced pressure to remove residual solvent. The obtained GD–ZnO nanocomposites were dried under a vacuum and stored in airtight containers for further analysis. To investigate the influence of composition on the structural and optical properties, samples were prepared with GD-to-ZnO weight ratios of 1:1, 1:2, and 1:0.5.

### 2.4 Characterization Techniques

Fourier-transform infrared (FT-IR) spectra were recorded on a PerkinElmer Spectrum Two spectrometer (PerkinElmer Inc., Waltham, MA, USA) using Attenuated total reflectance (ATR) mode in the 4000–500 cm^–1^ range. Ultraviolet–visible (UV–Vis) spectra were acquired with a Lambda 365 UV–Vis spectrophotometer (PerkinElmer Inc., Waltham, MA, USA) in 1 cm quartz cuvettes over the 200–800 nm range at room temperature under ambient conditions. Raman spectroscopy analyses were carried out using a LabRAM HR-EVO Horiba spectrometer (Horiba, Kyoto, Japan) equipped with a 532 nm laser, a 100× objective, and a CCD Syncerity Horiba detector (Horiba, Kyoto, Japan). Photoluminescence (PL) analyses were performed using a spectrofluorometer NanoLog modular (Horiba, Ltd., Kyoto, Japan) under excitation with a xenon lamp; the nanomaterials’ water dispersions were analyzed at the concentration of 100 ng/mL exciting the samples at the excitation wavelengths from 320 to 360 nm. Thermogravimetric analysis (TGA) was carried out using a PerkinElmer TGA 4000 instrument under an argon atmosphere (20 mL/min). Samples (1–2 mg) were placed in alumina crucibles and heated from 25 to 1000 °C at a rate of 10 °C/min. Zeta potential and size measurements were accomplished using the Zeta sizer 3000 instrument (Malvern). X-ray diffraction (XRD) patterns were recorded on a Bruker D2 diffractometer (Bragg–Brentano geometry) using Cu Kα radiation (λ = 1.5406 Å) over a 2θ range of 15–60° with a step size of 0.02° and a scan rate of 0.4°/min. Scanning electron microscopy (SEM) was performed using an FEI Quanta 450 microscope (Thermo Fisher Scientific, Waltham, MA, USA) in low-vacuum mode for morphological analysis. The Energy-Dispersive X-ray (EDX) analyses were made using an Octane plus Silicon Drift Detector (Ametek, Berwyn, PA, USA), equipped with 30 mm^2^ Super Ultrathin Windows (SUTWs). The specific surface area and porosity of the samples were evaluated by nitrogen adsorption–desorption measurements carried out at 77 K using a Quantachrome ASiQwin analyzer (Anton Paar Companies, Graz, Austria). The specific surface area was calculated using the Brunauer–Emmett–Teller (BET) method, while the pore size distribution was derived from the adsorption branch of the isotherms using the Barrett–Joyner–Halenda (BJH) model.

### 2.5 Sensor Fabrication and Gas Sensing Tests

Using alumina substrates (6 × 3 mm) with platinum interdigitated electrodes, thick films of GD–ZnO nanocomposites dispersed in water (1–10 mg/mL) and ZnO colloidal suspension in water were printed to fabricate the sensing devices. A Pt heater was integrated on the underside of the electrodes. Electrical measurements were carried out in the temperature range from room temperature to 350 °C under a synthetic dry air flow of 100 sccm. Resistance data were obtained by using the four-point probe method.

Gas sensing performance was evaluated by using a flow-through setup. A dual-channel power supply (Agilent E3632A) was used to bias the built-in heater, while a multimeter/data acquisition unit (Agilent 34970A) collected the resistance data. For H_2_S detection, pulses of the gas from certified cylinders were injected into the 5 mL test chamber at 100 sccm. Desired concentrations (0.125–4 ppm) were achieved by controlling the gas flow with mass flow controllers and using dry synthetic air (20% O_2_ in N_2_) as the carrier/diluent.

The gas response, *S*, is defined as

$$S=\frac{R_{0}}{R_{g}}$$

where *R*_0_ is the sensor resistance in synthetic air (baseline), and *R*_g_ is the resistance measured upon exposure to different H_2_S concentrations.

## 3 Results and Discussion

### 3.1 Chemical and Morphological Characterizations

The chemical strategy adopted in this work allows the controlled formation of GD–ZnO nanocomposites through a simple solution-phase approach designed to maximize interfacial contact while preserving the intrinsic properties of both components. By exploitation of the high surface reactivity and oxygen-functionalized edges of graphene dots, ZnO nanoparticles are homogeneously anchored without the need for additional surface modifiers or complex multistep functionalization procedures. The chemical composition of the synthesized materials was investigated by FT-IR spectroscopy (Figure 2). The FT-IR spectra of the three composites GDs–ZnO (green trace 1:1, blue trace 1:0.5, and pink trace 1:2) confirm the presence of the characteristic functional groups of ZnO and the successful incorporation of GDs in the conjugates. In the spectrum of pure ZnO (red trace), a broad absorption band centered at 3406 cm^–1^ corresponds to O–H stretching vibrations from surface-adsorbed water or hydroxyl groups, while the band observed at 511 cm^–1^ is assigned to the Zn–O stretching mode, indicative of the ZnO lattice. The FTIR spectrum of GDs (black trace) shows two peaks at 1721 and 3440 cm^–1^, which are related to the vibrations of the C=O and the O–H bonds, respectively. The two additional peaks at 1618 and 1072 cm^–1^ can be ascribed to the stretching of C=C bonds and C–O stretching of alkoxy groups. In the three GD–ZnO nanocomposites, there are bands at 1733 cm^–1^ (C=O stretching of carbonyl groups), 1587 cm^–1^ (C=C stretching of aromatic or graphitic domains), and 1070 cm^–1^ (C–O or C–O–C stretching). The coexistence of these carbon-related bands, along with the slight shift and intensity variation of the O–H stretching region compared with pure ZnO, provides strong evidence of GD incorporation and possible interfacial interactions (e.g., hydrogen bonding or charge transfer) between GDs and ZnO. The persistence of the Zn–O vibration at ∼511 cm^–1^ indicates that the ZnO crystal structure remains intact after GD integration.

**Figure 2:**
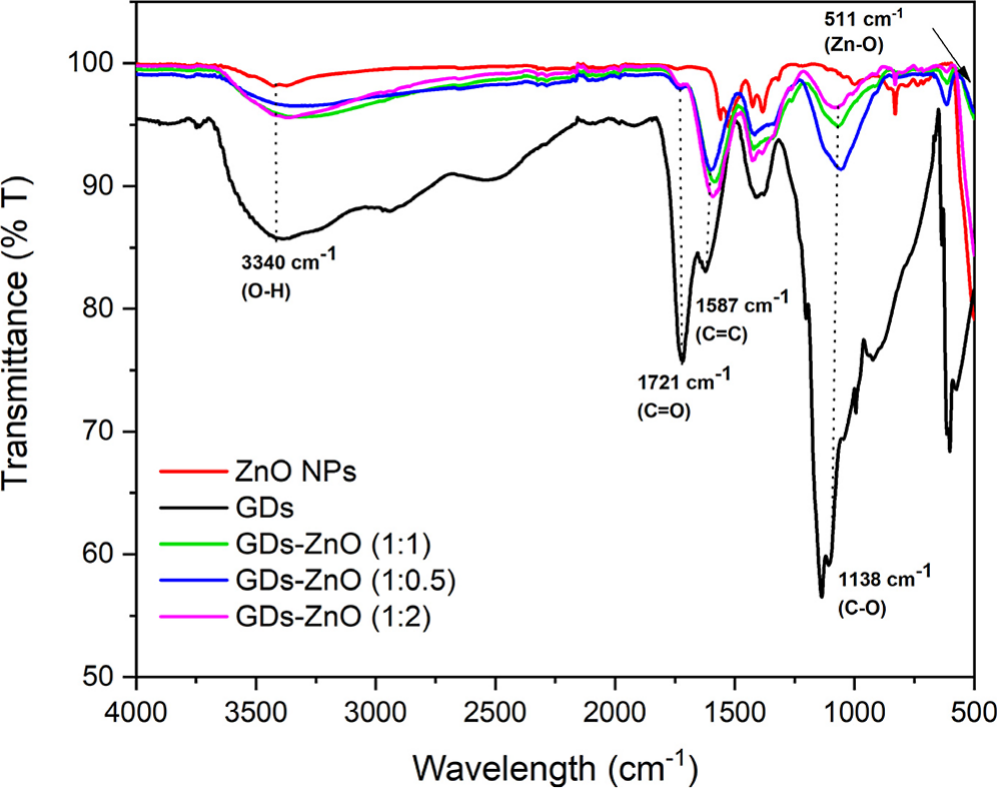
FT-IR spectra of pure ZnO nanoparticles (red trace), the pristine GDs (black trace), and GD–ZnO nanocomposites at different weight ratios (green trace 1:1, blue trace 1:0.5, and pink trace 1:2).

The UV–vis absorption spectra of pure ZnO, pristine GDs, and GD–ZnO nanocomposites at different weight ratios are shown in Figure 3. Pristine ZnO exhibits a sharp absorption edge centered around 370 nm, corresponding to its near-band-edge transition. In contrast, the GD–ZnO nanocomposites display a broadened and slightly red-shifted absorption extending into the visible region (400–500 nm), which can be attributed to the π–π* transitions of the sp^2^-hybridized carbon domains in the GDs and to interfacial electronic coupling between ZnO and GDs. The coexistence of the characteristic ZnO absorption edge and the additional visible-light absorption confirm the successful formation of the hybrid nanocomposite and its enhanced light-harvesting capability. To further analyze the effect of GD incorporation on the electronic structure, the optical band gap energies were estimated using the Tauc method assuming a direct allowed transition, according to the relation:

$${\left(\alpha h\nu \right)}^{2}=A\left(h\nu -E_{g}\right)$$

where α is the absorption coefficient, *h*ν is the photon energy, *A* is a constant, and *E*_g_ is the optical band gap. The corresponding Tauc plots are provided in the Supporting Information (Figures S1–S3). The extracted band gap values are approximately 3.25 eV for pristine ZnO, 2.56 eV for GDs, and 2.90 eV for the GD–ZnO nanocomposite, indicating a clear band gap narrowing upon hybridization. Such spectral modifications suggest efficient electronic interaction between the ZnO conduction band and the π-conjugated system of GDs, which facilitates interfacial charge transfer. This enhanced charge transport is advantageous for surface reaction kinetics and plays an important role in improving gas-sensing performance.

**Figure 3:**
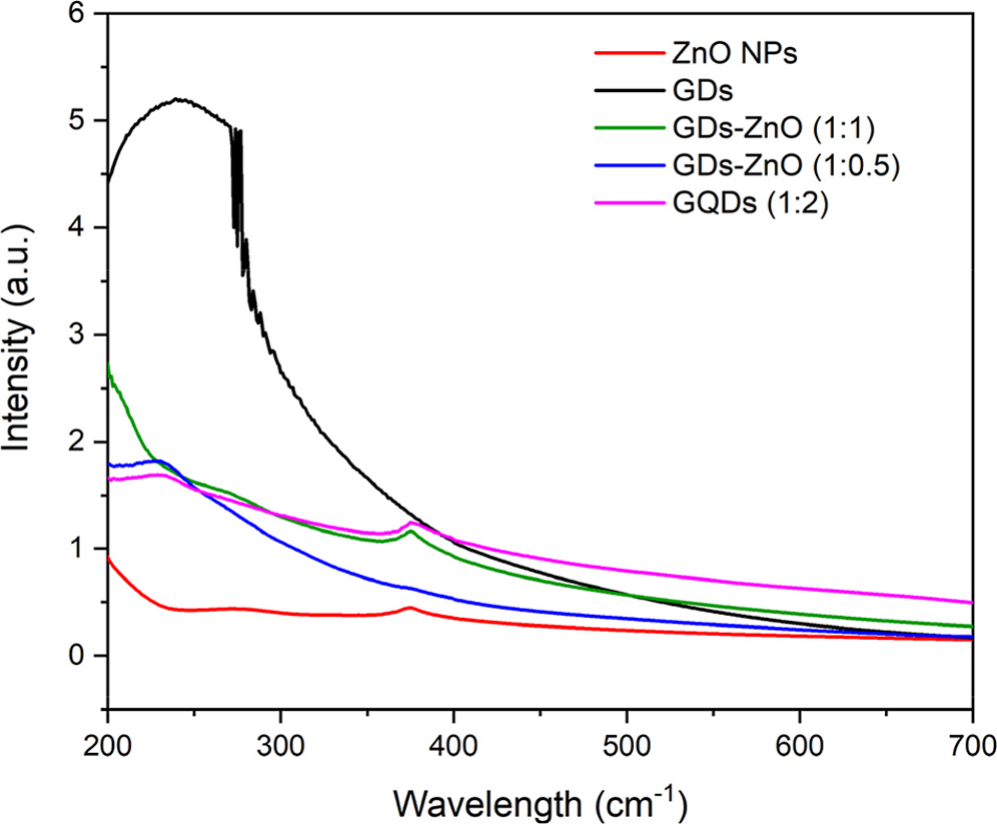
UV–Vis absorption spectra of ZnO NPs (red), pristine GDs (black), and GD–ZnO nanocomposites at different weight ratios (blue 1:0.5, green 1:1, and pink trace 1:2).

Figure 4 illustrates the Raman spectra of GDs (red line) and GD–ZnO nanocomposites (black line) deposited on a silicon substrate, recorded under 532 nm laser excitation, and ZnO spectrum (green line), got from the “RamanLife” Raman Spectra Database and used as a reference [RamanLife Database. https://ramanlife.com/library/zinc-oxide/]. Regarding the GD samples, they show the typical characteristic bands of carbon-based materials under visible-light excitation, located between 1330 and 1360 cm^–1^ and 1560–1600 cm^–1^, known as the D and G bands, respectively.^29^ The GD sample exhibits well-defined D (∼1359 cm^–1^) and G (∼1587 cm^–1^) bands, characteristic of disordered or partially ordered sp^2^ carbon domains. A redshift and broadening of the above-mentioned bands are evident in the GD–ZnO sample, possibly due to a modification of the sp_2_ conjugation and to a vibrational enhancement with ZnO–carbonaceous edge coupling and/or greater activation of the A_1_g modes.^30,31^ As for the E_2_ modes of ZnO (around ∼437 cm^–1^), it is evident that we have a shift and broadening in the GD–ZnO sample compared to what is shown in the ZnO-only spectrum, suggesting a change in the ZnO structure because of the interaction with GDs.^31^ Note that the fundamental Raman band of single crystal silicon (∼520 cm^–1^) is also reported in Figure 4 for the sake of completeness.

**Figure 4:**
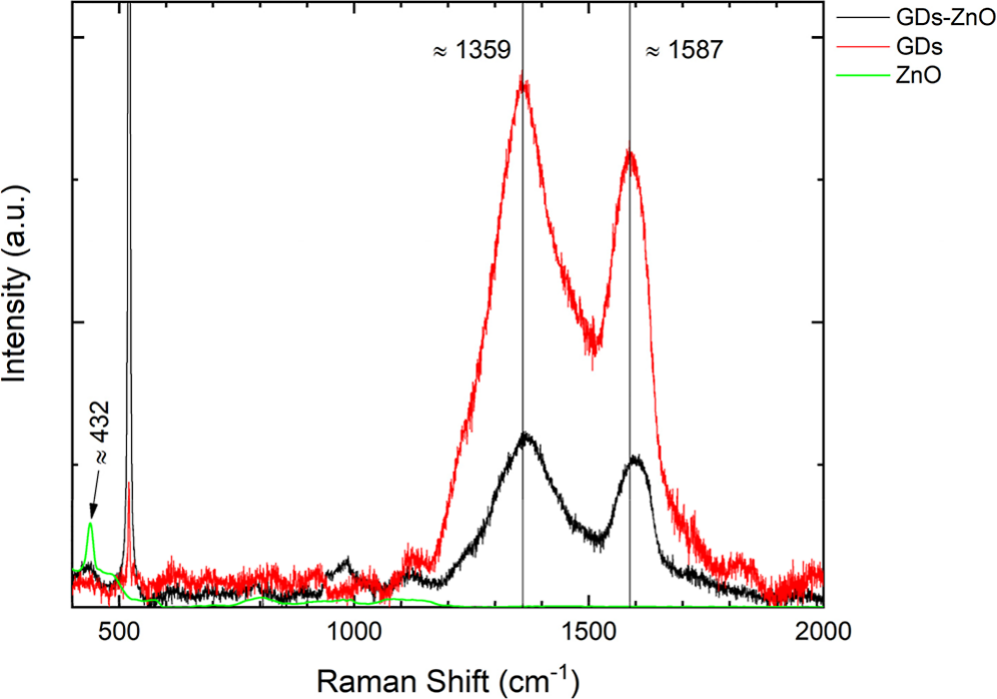
Raman spectra of GDs (red) and GD–ZnO nanocomposites (black), as deposited onto silicon substrate, and of ZnO (green line).

The PL properties of nanomaterials provide valuable insight into their size, surface functionalization, and structural features, which play a crucial role in determining their performance in sensing, imaging, and catalytic applications. In this study, the excitation wavelength was fixed at 360 nm, a typical excitation condition for carbon-based nanomaterials.^32^ Figure 5 shows the PL spectra of GDs, ZnO nanoparticles, GDs, and GD–ZnO nanocomposites with different weight ratios. GDs exhibit a broad visible emission centered at ∼510–520 nm, originating from quantum confinement and surface-state recombination. ZnO nanoparticles display a dominant visible emission around ∼580–600 nm, typically associated with deep-level defect states. Upon hybridization, the GD–ZnO composites show a clear redshift of the emission peak accompanied by a significant decrease in PL intensity, with the GD–ZnO (1:0.5) sample exhibiting the strongest quenching. This behavior indicates strong interfacial coupling and efficient charge transfer between ZnO and the π-conjugated domains of GDs, leading to suppressed radiative recombination. The composition-dependent PL quenching confirms the formation of an effective electronic junction at the ZnO–GD interface, which is expected to enhance the surface charge availability and gas-sensing performance.

**Figure 5:**
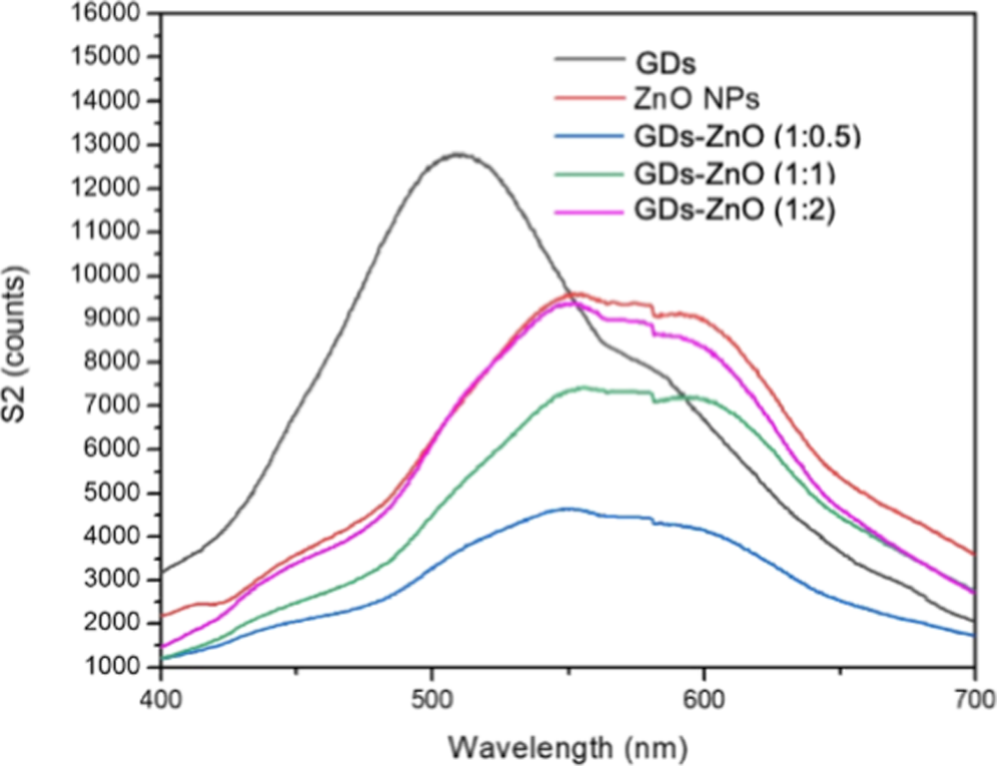
PL spectra of ZnO NPs (red), pristine GDs (black), and GD–ZnO nanocomposites at different weight ratios (blue 1:0.5, green 1:1, and pink 1:2), in deionized water, at the excitation wavelength of 360 nm (concentration of 100 ng/mL).

The hydrodynamic behavior and surface charge of GDs, ZnO nanoparticles, and the corresponding GD–ZnO nanocomposites were investigated through DLS and zeta potential measurements (Figures 5 and 6). The DLS intensity profile of pristine GDs revealed a sharp monodisperse population centered at 4.8 nm, confirming their ultrasmall dimensions and excellent dispersibility in water. Conversely, ZnO nanoparticles showed a broad distribution spanning 150–400 nm with a main peak in the 200–250 nm range, indicative of extensive aggregation in an aqueous medium. The hybrid GD–ZnO systems exhibited intermediate hydrodynamic behavior that varied systematically with composition. The 1:0.5 formulation (GQD-rich) displayed a primary peak centered around 100–130 nm, significantly smaller than pristine ZnO and consistent with the modulation of nanoparticle association by the high GD content. The 1:1 composite presented a broader distribution with a maximum between 150 and 200 nm, approaching the ZnO profile but still showing a measurable reduction in hydrodynamic size. Finally, the 1:2 formulation (ZnO-rich) exhibited the largest hydrodynamic diameters among the hybrids (220–300 nm), closely overlapping the ZnO curve and confirming the increasingly dominant contribution of the oxide. Zeta potential measurements further supported this compositional trend. GDs exhibited a strongly negative surface charge (≈−40 mV), consistent with the presence of deprotonated oxygenated groups. ZnO nanoparticles showed a positive zeta potential (≈+14 mV) at neutral pH, in line with their amphoteric nature and surface protonation state. The GD–ZnO hybrids displayed intermediate ζ-values: the 1:0.5 sample remained negative (≈−25 mV), the 1:1 formulation showed moderately negative values (≈−15 mV), whereas the 1:2 composite shifted toward positive charge (≈+5 mV). Taken together, the DLS and zeta potential data demonstrate that increasing the ZnO content progressively leads to larger hydrodynamic sizes and shifts the surface charge toward more positive values, whereas GD-rich formulations retain smaller dimensions and more negative ζ-potentials. This confirms a clear, composition-dependent interaction between GDs and ZnO surfaces.

**Figure 6:**
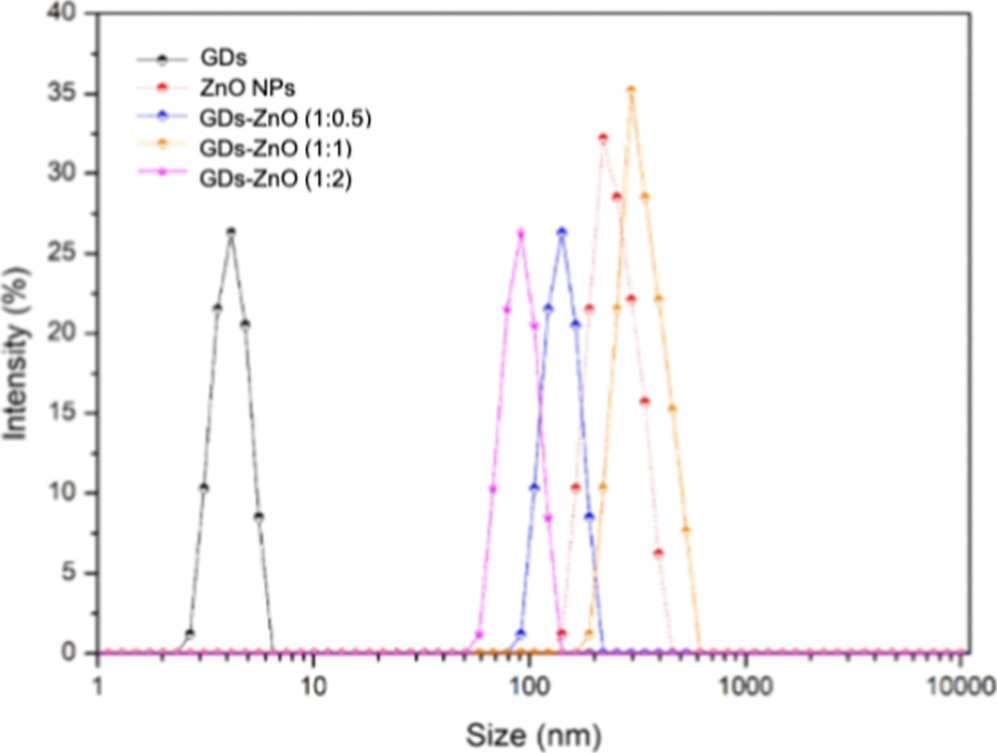
Size distribution by intensity obtained from DLS analysis for GDs, ZnO nanoparticles, and GD–ZnO nanocomposites at different weight ratios (1:0.5, 1:1, and 1:2).

The thermogravimetric behavior of pure ZnO, pristine GDs, and GD–ZnO nanocomposites with different GD-to-ZnO weight ratios was investigated to assess their thermal stability and to further confirm the formation of the hybrid structures (Figure 7). The TGA curve of pure ZnO exhibits excellent thermal stability, with only a minor weight loss (<2%) below 200 °C, attributed to the desorption of physically adsorbed water and surface hydroxyl groups. No significant decomposition occurs up to 1000 °C, confirming the thermal robustness of ZnO. In contrast, the GD sample shows a more pronounced multistep degradation pattern. The initial weight loss below 150 °C is related to the removal of surface-bound water and labile oxygen-containing groups, followed by a major weight loss between 250 and 500 °C due to the thermal decomposition of carbonaceous domains and oxidation of graphitic carbon. The complete degradation of GDs occurs around 600 °C, leaving a minimal residual mass. The GD–ZnO composites exhibit intermediate thermal behavior dependent on ZnO content. The 1:0.5 sample shows the largest weight loss, reflecting its higher GD fraction, while the 1:1 composite displays slightly higher stability, likely due to better dispersion and interfacial interactions at lower ZnO loading. The 1:2 sample, with the highest ZnO content, retains the most residual mass, reflecting the stabilizing effect of the oxide. Overall, the higher residual mass of all GD–ZnO composites confirms successful ZnO incorporation and strong interfacial coupling, supporting their suitability for high-temperature sensing applications.

**Figure 7:**
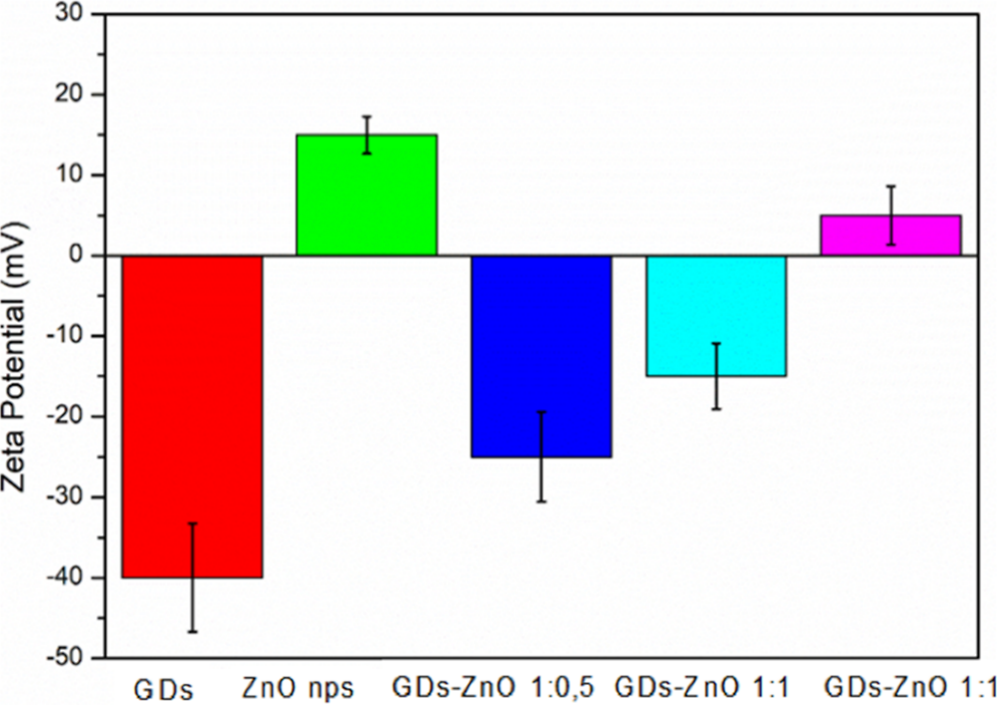
Zeta potential values of GDs, ZnO nanoparticles, and GD–ZnO nanocomposites.

XRD measurements of GDs and the GD–ZnO nanocomposite (1:1) as a representative example to investigate its crystal structure and phase integrity were performed in water solutions and as solid residues after solvent evaporation to investigate the differences between the GDs and the GD–ZnO nanocomposites. We also measured the XRD pattern of the ZnO nanocomposite added using a standard Powder Sample Holder. The precipitate solid samples were obtained by the drop-cast method of solutions of GDs and GD–ZnO on a [100] Si wafer substrate and used for the measure after complete drying. In the case of liquid samples, we made use of a homemade Airtight Sample Holder in which the GDs and the GD–ZnO water solutions were sealed by means of Kapton gasket to avoid solvent evaporation. Note that, in this latter case, performing XRD measurements on the ZnO nanopowder dispersed in water is not ideal, as the signal from water would dominate, and the ZnO would no longer be in its crystalline form. The XRD pattern of investigated samples is shown in Figure 8, with an insert for samples in solution, after background subtraction. In Figure 8 are reported (i) ZnO nanopowder, (ii) graphite, and ZnO crystals’ pattern along with Miller indices. As for precipitate GDs (solid sample, blue line), we observe a broad diffuse bump from 23 to 35 °C (002 plane in graphite, see graphite reference in Figure 8 reported as a black line), that evidence the presence of graphene dots characterized by a tighter layer stacking.^33^ Also, the presence of a well-defined peak around 32° and a broader peak at 28.5° indicate a more graphitic packing.^34^ A second very broad band around ∼40–45° is present indicating poor in-plane order and very lateral coherence lengths.^35^

**Figure 8:**
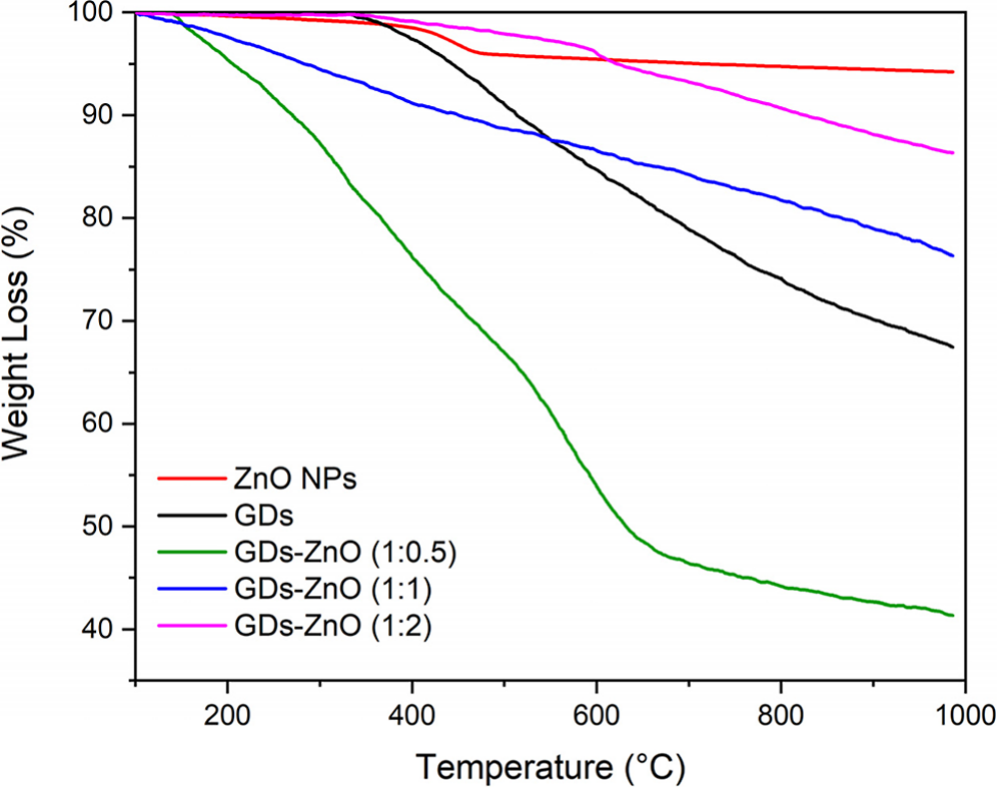
TGA curves of pure ZnO, the pristine GDs, and GD–ZnO nanocomposites at different weight ratios (blue 1:0.5, green 1:1, and pink 1:2).

In the case of the ZnO complexed sample, we observe a shift of the first broad peak toward lower angle and a depression of the indicating an expansion of the interlayer spacing. Moreover, some additional narrow peaks appear in the XRD pattern at angles of 31.7°, 34.4°, 36.2°, and 47.5° that can be related to ZnO residual salts (planes: 100, 002, 101, 102). We also observe the presence of few peak characteristics of graphite and intercalated graphite oxide at 26°, 42°, and 13°.^36^ The narrow peak at 33° can instead be ascribed to sodium sulfate- and nitrate-precipitated phases developed in sample processing. The equivalent spectra taken from samples in water solution resemble the behavior of the solid counterparts, evidencing the attenuation of both broad peaks in the complexed sample corroborating the hypothesis of an integration of GDs with ZnO nanopowder.

We can obtain additional information on grain size and strain by analyzing the peak half-width.^37^ By applying the Scherrer formula to the graphite peak at 28.5° for both samples, we found that in the absence of ZnO, the graphene grain size is approximately 3.87 Å; in contrast, in the nanocomposite sample, this size is much larger (around 26.19 Å), indicating graphene stacking. Regarding ZnO, we identified several diffraction peaks and computed the average crystallite size and strain using the Williamson–Hall (WH) Plot approach.^38^ This technique relies on the fact that the approximate expressions for size broadening, β_l_, and strain broadening, β_e_, show distinctly different dependencies on the Bragg angle, θ^39^:

$$\beta_{tot}\cos\theta =C\varepsilon \sin\theta +\frac{K\lambda }{L}$$

We analyzed the widths of five ZnO peaks, obtaining the results reported in Table S1. The WH Plot is shown in Figure 9 together with the linear fit (Figure S4), computed from the equation above. From the fitting results displayed in the same figure, we determined an average grain size of 84.78 Å and a strain coefficient of approximately 0.038. These values appear to be somewhat higher when compared to the ZnO nanopowder data reported in the reference paper.^39^

**Figure 9:**
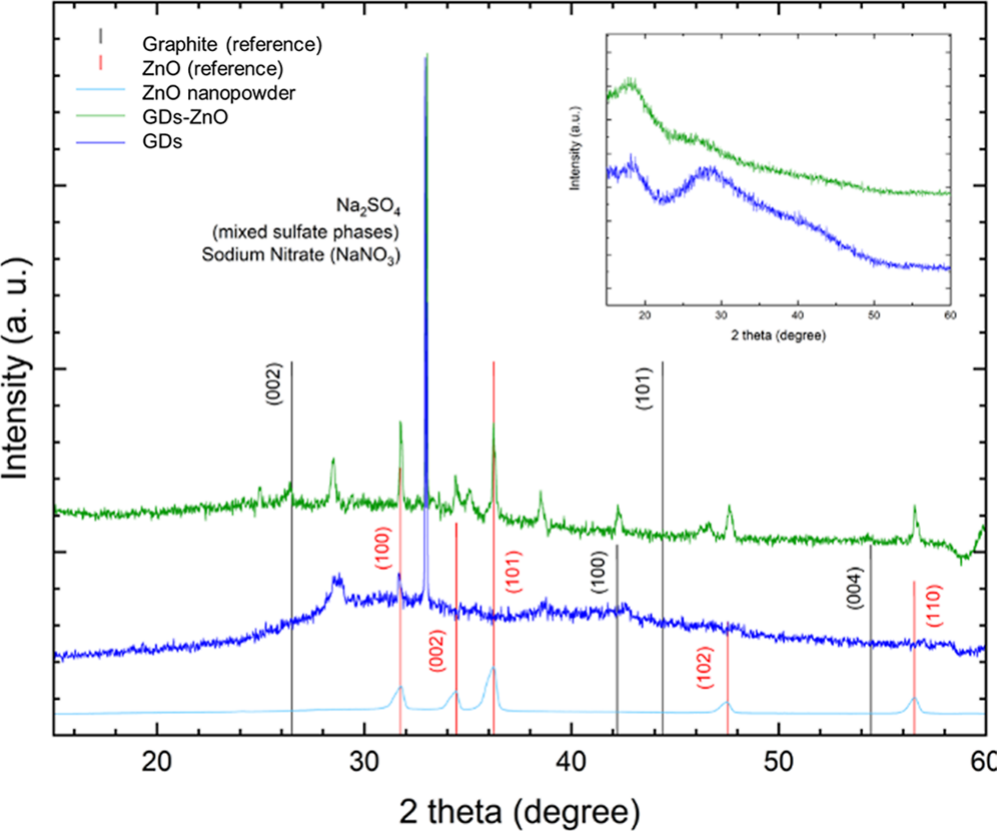
XRD patterns of the pristine GDs (blue line), GD–ZnO nanocomposite (green line), and ZnO NPs (light blue line); references for graphite and ZnO with their Miller indices are included. In the inset are reported the XRD spectra taken from samples in the water solution.

To complement the structural analysis, SEM was employed to examine the surface morphology and microstructural features of the pristine ZnO and GD–ZnO (1:1) nanocomposite, selected as representative samples (Figure 10). The pristine ZnO sample (Figure 10A) exhibits a granular and highly agglomerated morphology consisting of quasi-spherical nanoparticles. The surface is rough and porous, indicative of a large specific surface area, which is advantageous for gas-sensing applications by facilitating gas diffusion and enhancing surface reactivity. In contrast, the GD–ZnO nanocomposite (Figure 10B) displays a more dispersed morphology with reduced agglomeration. Although the ZnO NPs retain a predominantly granular structure, the incorporation of GDs suppresses particle clustering and results in a finer and more homogeneous surface texture. This more open microstructure is expected to promote improved adsorption–desorption kinetics during gas sensing, thereby contributing to the enhanced sensing performance. These observations are consistent with the EDX elemental analysis, which confirms the homogeneous distribution of Zn, O, and cations without detectable impurities, supporting the successful integration of GDs within the ZnO matrix.

**Figure 10:**
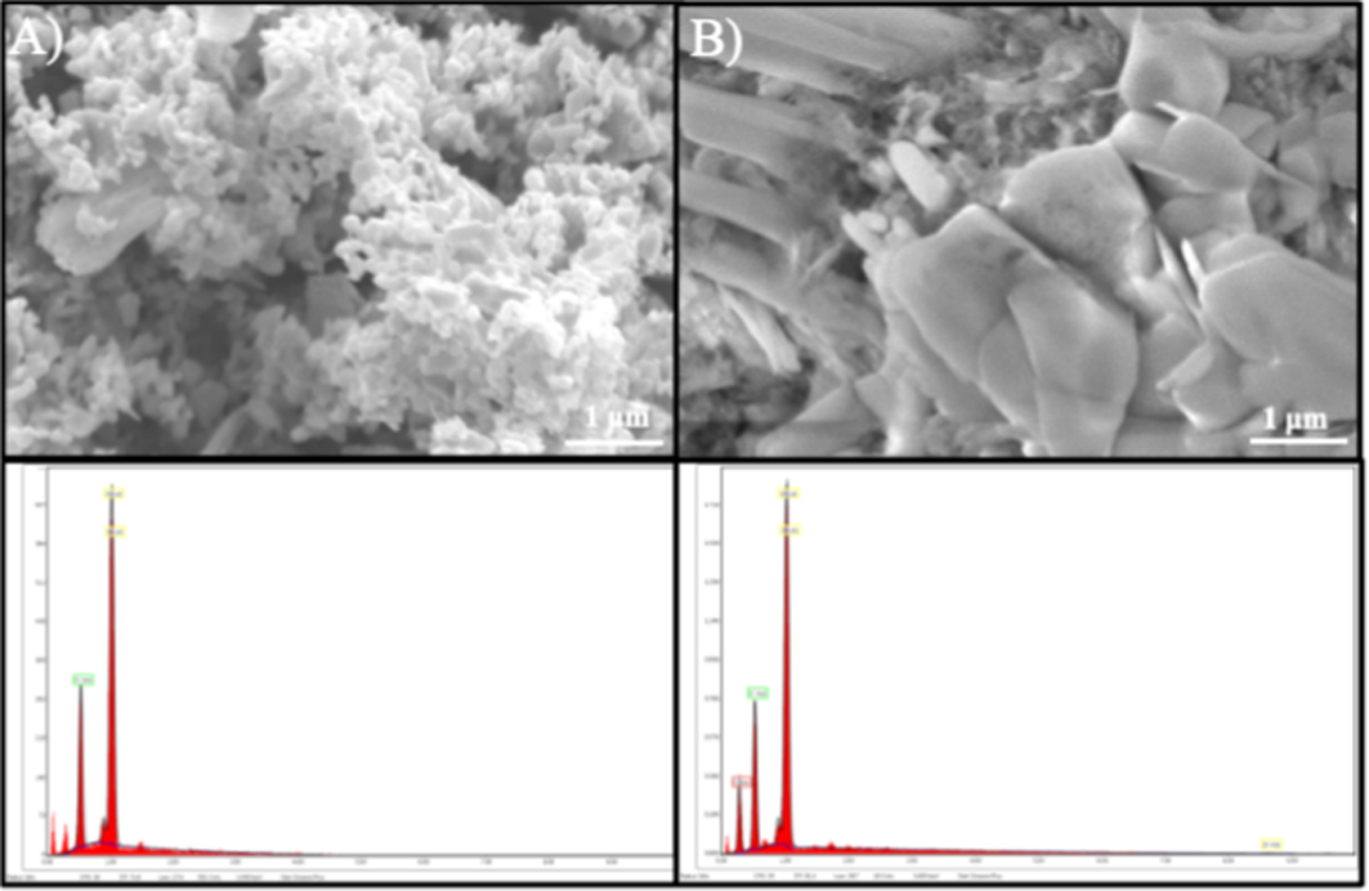
SEM micrographs of (A) pure ZnO NPs and (B) GD–ZnO nanocomposite (ratio 1:1) consistent with EDX elemental analysis.

BET analysis was employed to evaluate the porosity and specific surface areas of the synthesized materials. The pore size distribution of pristine ZnO and the GD–ZnO composite, determined using the BJH method, shows average pore diameters of 17.6 and 7.1 nm, respectively. The incorporation of GDs into the ZnO matrix leads to a noticeable narrowing of the pore size distribution in the composite, in good agreement with literature reports on ZnO–graphene-based systems.^40^ In addition, the specific surface area increases from 16.2 m^2^/g for pure ZnO to 25.5 m^2^/g for the GD–ZnO composite, confirming that GD incorporation promotes a more open and accessible mesoporous structure.

### 3.2 Gas Sensing Performance

Dynamic response curves of the pure ZnO and GD–ZnO (ratio 1:1) at a concentration range of 0.125 to 4 ppm of H_2_S are reported in Figure 11A. All gas-sensing measurements were performed in a closed chamber using dry synthetic air (20% O_2_ balanced with N_2_) as the carrier gas, allowing the intrinsic sensing behavior of the materials to be evaluated under humidity-free and well-controlled conditions. As shown in Figure 11B, the maximum response was observed at 200 °C, which was selected as the optimal operating temperature for subsequent measurements. This trend can be explained by the competition between thermally activated surface reactions, which dominate at moderate temperatures, and rapid desorption of gas molecules at elevated temperatures, which lowers the surface coverage. The GD–ZnO sensor achieved its optimal response at this intermediate temperature range, confirming the beneficial role of GDs in enhancing adsorption–desorption dynamics and charge transport. For the tested samples, a clear decrease in resistance was observed upon H_2_S exposure, followed by full recovery to the baseline once the gas was removed. This behavior confirms that both ZnO and GD–ZnO sensors maintain the typical n-type conduction characteristics of ZnO during H_2_S sensing. However, the GD–ZnO nanocomposite sensor shows a significantly higher response intensity compared with pure ZnO (Figure 11C). The quantified response and recovery time amount to approximatively 90 and 250 s, respectively. This enhancement can be attributed to the synergistic interaction between ZnO and GDs, which increases the number of active adsorption sites and facilitates charge transfer processes at the heterojunction interfaces. The introduction of GDs improves the surface-to-volume ratio and electronic conductivity, promoting more efficient electron exchange between the adsorbed oxygen species and the target gas molecules. The presence of graphitic domains in GDs likely facilitates rapid electron transport across the sensing layer, leading to an amplified modulation of resistance during gas exposure. Moreover, the mild redshift observed in the UV–Vis spectra of GD–ZnO supports the formation of an interfacial electronic coupling that enhances charge carrier mobility—consistent with the superior sensing performance.

**Figure 11:**
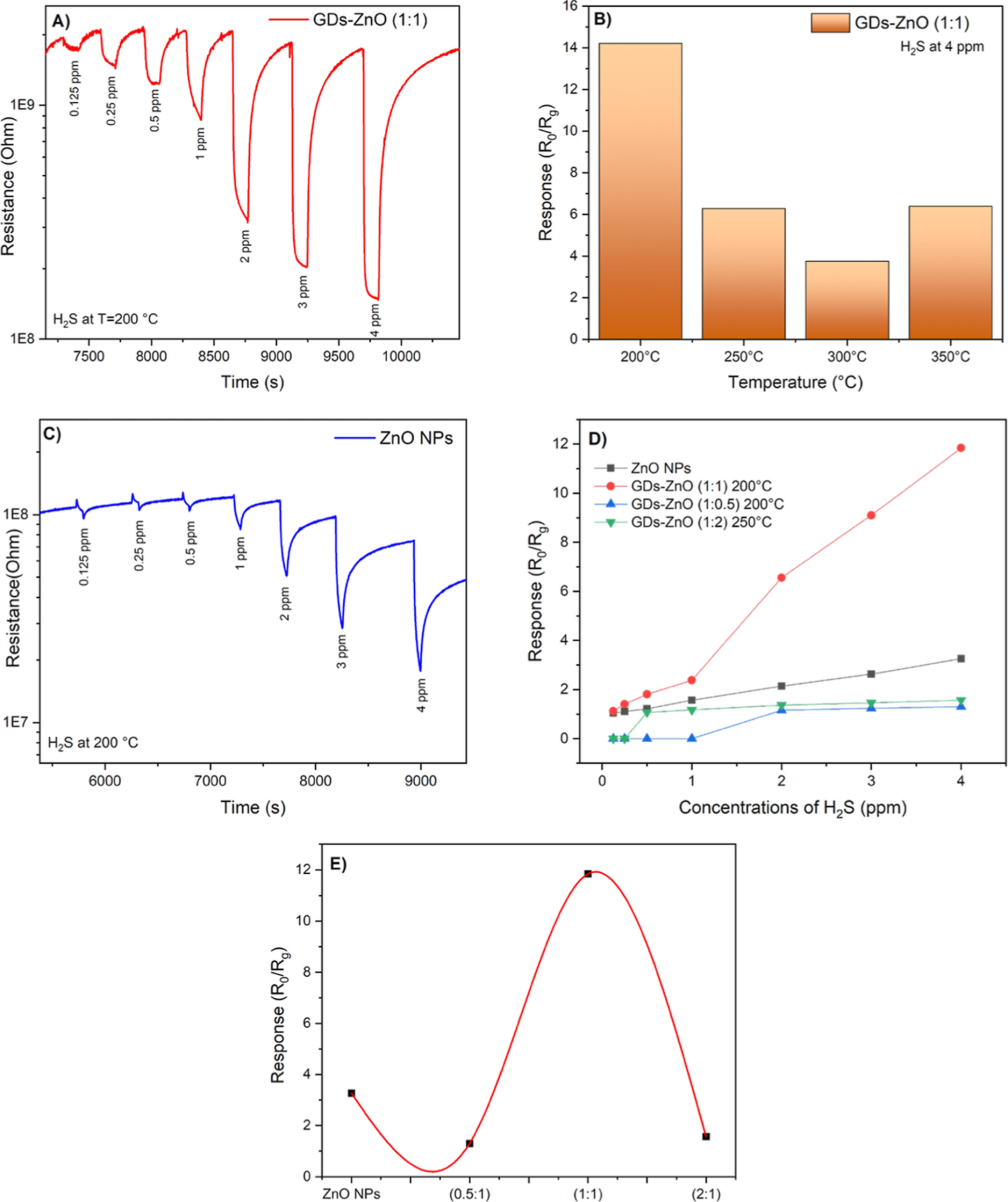
Gas sensing performance of the GD–ZnO sensor toward H_2_S: (A) dynamic response at various concentrations, (B) temperature-dependent response, (C) response of pure ZnO, (D) calibration curve of all the sensors, (E) gas response as a function of ZnO and GD ratios.

To evaluate the effect of composition on gas-sensing performance, GD–ZnO nanocomposites with different GD-to-ZnO molar ratios were compared with pristine ZnO (Figure 11D). A single calibration plot correlating the sensor response (*S* = *R*_0_/*R*_g_) with the H_2_S concentration was obtained for all samples. The GD–ZnO (1:1) sensor operated at 200 °C exhibits a clear linear response over the investigated concentration range, described by the equation *S* = 0.448 + 2.858*C* with a high correlation coefficient (*R* ^2^ = 0.995). In contrast, the GD–ZnO (1:0.5) sensor at 200 °C and the GD–ZnO (1:2) sensor at 250 °C show significantly lower responses and a tendency toward saturation at higher concentrations. The sensor response exhibits a volcano-type dependence on the GD–ZnO ratio, reaching a maximum at 1:1 composition (Figure 11E). Our results indicate that the incorporation of GDs effectively reduces ZnO nanoparticle agglomeration and introduces a high density of edge-rich, defect-active sites, which act as additional adsorption centers for gas molecules. Moreover, the improved dispersion of ZnO in the GD matrix facilitates gas diffusion throughout the sensing layer. The superior performance of the 1:1 nanocomposite is attributed to the synergistic interaction between ZnO nanoparticles and GDs. GDs provide electron-rich surface states that facilitate charge transfer, while well-dispersed ZnO nanoparticles supply active oxygen species for H_2_S adsorption, promoting efficient heterojunction formation. Conversely, excess ZnO in the 1:2 composite likely induces particle agglomeration and reduced interfacial contact, requiring higher operating temperatures to achieve measurable responses. These results are consistent with literature reports indicating that pristine ZnO-based sensors typically require elevated operating temperatures.^41,42^ The fitting parameters are summarized in Table 1, where the slope value (2.858 ± 0.133) represents the sensitivity of the GD–ZnO (1:1) sensor, confirming its enhanced response toward H_2_S at 200 °C.

**Table 1:** Linear Fitting Parameters Obtained from the Calibration Curve of the GD–ZnO Sensor toward H_2_S at 200 °C

parameter	GDs–ZnO (1:1)
optimal temperature (°C)	200
linear range (ppm)	0.125–4 ppm
sensitivity (ppm^–1^)	2.8582 × 10^–3^
*R* ^2^	0.995

The repeatability and stability of the GD–ZnO sensor were evaluated through consecutive H_2_S exposure cycles (Figure 12). The nearly identical response and recovery curves recorded for multiple cycles confirm the excellent reproducibility and reversibility of the sensing behavior. Moreover, the baseline resistance remained stable over time, suggesting that the nanocomposite exhibits strong structural and chemical stability under repeated gas exposure.

**Figure 12:**
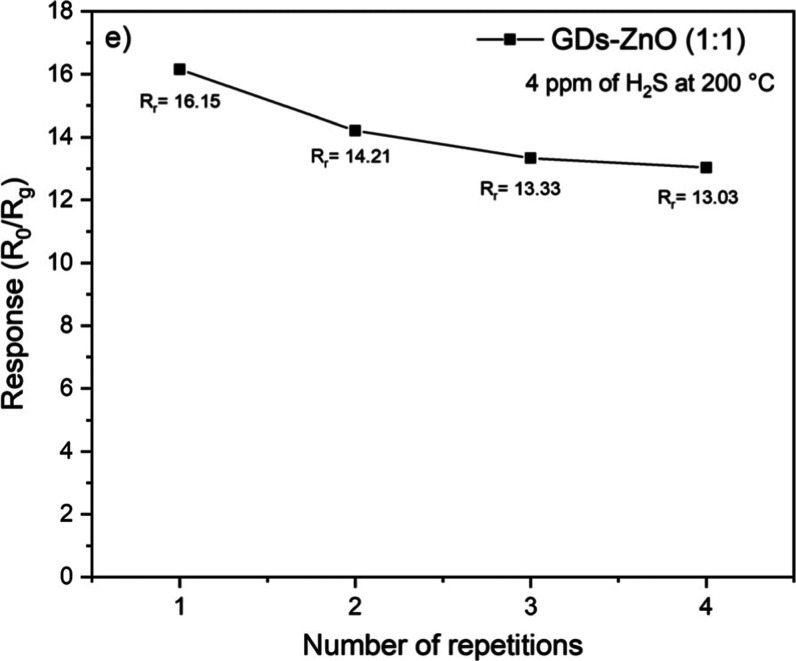
Repeatability of the GD–ZnO (1:1) sensor toward H_2_S at 200 °C, showing stable response over repeated cycles.

According with the data reported in the literature, Table 2 allows a clear comparison between conventional metal oxide-based sensors and those incorporating graphene-based nanostructures. Materials without graphene, such as ZnO/SnO_2_ porous nanocomposites^42^ or Ni-doped ZnO nanowires,^43^ generally exhibit high responses but require elevated operating temperatures (250–325 °C). In contrast, composites containing graphene derivatives, such as the SnO_2_/rGO composite,^44^ achieve significant responses while operating at lower temperatures, including room temperature in some cases. The GD–ZnO (1:1) sensor developed in this work exhibits several notable strengths, particularly in terms of range of concentrations, linearity, and operating conditions. This combination of low detection concentrations, high linear correlation, and relatively lower temperature stands as a strength versus many metal-oxide-based sensors that require >300 °C or achieve linear response only at higher ppm. Moreover, the slope (2.8582 response ppm^–1^) is competitive, meaning that the sensor delivers strong sensitivity even at low concentrations.

**Table 2:** Comparative Table of Sensing Parameters

material	*T* (°C)	concentration range (ppm)	response (*R*_0_/R_g_)	response time (s)	recovery time (s)	sensitivity (ppm^–1^)	ref
GD–ZnO (1:1) nanocomposite	200	0.125–4	11.88	90	250	2.8582 × 10^–3^	(present work)
ZnO/SnO_2_ heterogeneous nanospheres	300	0.05–100	99.6	30	-	-	^41^
ZnO/SnO_2_ porous nanocomposite (3:4)	325	0.5–5	9.7	17	8835	-	^42^
Ni-doped ZnO nanowire arrays (8% Ni)	250	0.5–10	68.9	75	54	-	^43^
SnO_2_ quantum wire/rGO composite	RT	50	33	2	292	-	^44^
CNT/SnO_2_/CuO composite	RT	10–90	-	250	-	-	^45^

The selectivity of the GD–ZnO-based sensor was investigated toward various interfering gases, including CO, SO_2_, NO_2_, and H_2_, under identical experimental conditions at 200 °C. The nominal concentrations correspond to the certified maximum values of the gas cylinders, namely, 4 ppm for H_2_S, 80 ppm for CO, 16 ppm for SO_2_, 8 ppm for NO_2_, and 80,000 ppm for H_2_. As shown in Figure 13, the GD–ZnO sensor exhibited a markedly higher response to H_2_S (*S* = 14.2 at 4 ppm) compared to CO (*S* = 1.5), SO_2_ (*S* = 1.1), NO_2_ (*S* = 1.1), and H_2_ (*S* = 2.2).

**Figure 13:**
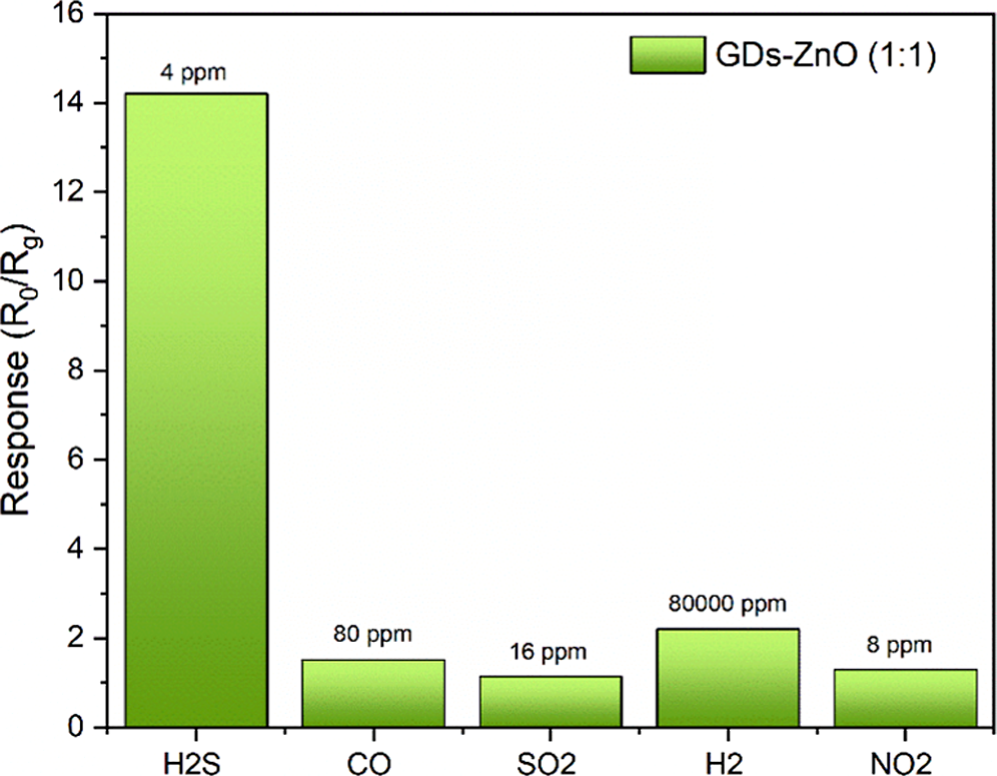
Selectivity of the GD–ZnO sensor toward various gases (H_2_S, CO, SO_2_, NO_2_, H_2_) at 200 °C.

Such behavior confirms the strong affinity of the GD–ZnO nanocomposite for H_2_S molecules, likely due to the high surface activity of the GDs and the efficient charge transfer occurring at the GD–ZnO heterointerface. This synergistic interaction enhances the adsorption and reaction of H_2_S species while minimizing cross-sensitivity to other reducing or oxidizing gases.

### 3.3 Gas Sensing Mechanism

The enhanced sensing performance of the GD–ZnO nanocomposite toward H_2_S can be explained based on the surface adsorption–desorption mechanism typical of n-type metal oxide semiconductors independent of the organic nature of the GDs.^46^ In ambient air, oxygen molecules adsorb on the ZnO surface and capture free electrons from the conduction band, forming chemisorbed oxygen species such as O_2_^–^, O^–^, and O^2–^ depending on the operating temperature, according to the following reactions:

$$O_{2}\left(gas\right)\to O_{2}\left(ads\right)$$

$$O_{2}\left(ads\right)+e^{-}\to O_{2}^{-}\left(ads\right)$$

$$O_{2}^{-}\left(ads\right)+e^{-}\to {2O}^{-}\left(ads\right)$$

When H_2_S gas is introduced, it reacts with the adsorbed oxygen species on the surface of ZnO, releasing the trapped electrons back into the conduction band, leading to a sharp decrease in resistance:

$$H_{2}S+{3O}^{-}\left(ads\right)\to H_{2}O+{\text{SO}}_{2}+{3e}^{-}$$

This electron replenishment enhances the conductivity of the sensing film.^47^

In the GD–ZnO nanocomposite, the presence of GDs introduces additional electronic pathways and active surface sites that promote a more efficient charge transfer and gas adsorption. The π-conjugated domains of GDs facilitate electron mobility, while the formation of a GD/ZnO heterojunction improves the separation of charge carriers. The built-in electric field at the interface enhances electron transfer from GDs to ZnO, amplifying the resistance modulation upon H_2_S exposure.

Overall, the intimate interfacial contact between ZnO and GDs increases the density of chemisorbed oxygen species, which further contributes to the improved sensitivity and faster response/recovery behavior observed experimentally.

## 4 Conclusions

In summary, GD–ZnO nanocomposites were successfully synthesized and systematically characterized by FT-IR, UV–vis, Raman, PL, XRD, SEM/EDX, BET, DLS, and TGA analyses, confirming the effective incorporation of ZnO nanoparticles within the GD matrix. The morphological and structural results evidenced a homogeneous dispersion and intimate interfacial contact between ZnO and GDs, which enhanced the electronic coupling and surface reactivity.

Gas sensing tests demonstrated that the GD–ZnO device exhibits a highly selective and reproducible response toward H_2_S at an optimal operating temperature of 200 °C, with a rapid resistance drop upon gas exposure and full recovery in synthetic air. Among the different compositional ratios studied (1:0.5, 1:1, and 1:2), the 1:1 GD–ZnO nanocomposite showed the highest sensitivity and best linear response in the 0.125–4 ppm concentration range (*R* ^2^ = 0.9946), maintaining fast response and recovery times. Moreover, the sensor displayed a pronounced selectivity toward H_2_S compared to those of CO, SO_2_, NO_2_, and H_2_, even when the latter were tested at much higher nominal concentrations. The enhanced performance is attributed to the synergistic interaction between ZnO and GDs, promoting efficient charge transfer and facilitating surface adsorption of H_2_S molecules. In this work, gas-sensing tests were carried out under controlled conditions using dry synthetic air in order to assess the intrinsic sensing properties of the proposed materials without the interference of humidity. Although these results represent a preliminary evaluation, future studies will be devoted to investigating the influence of humidity and real operating environments on the sensor performance.

These findings highlight the potential of GD–ZnO nanocomposites as efficient, low-cost materials for the development of high-performance H_2_S sensors operating under mild conditions, paving the way for future applications in environmental monitoring and industrial safety.

## Supplementary Material



## Notes

Tauc plots of ZnO NPs, GDs, and GDs–ZnO; WH plot of the ZnO peaks for the GD–ZnO nanocomposite; and data computed for the ZnO peaks by using the Williamson–Hall method (PDF)
