# Supplementary material for: Graphene Dot–ZnO Hybrid Nanostructures as High-Performance Chemiresistive Sensors for H2S Detection
Source: ACS Omega. 2026 Apr 10;11(15):23188–99. doi: 10.1021/acsomega.5c13473 (PMC13103760; doi:10.1021/acsomega.5c13473)
Supplement: Supplementary file 1 [file ao5c13473_si_001.pdf]

Supplementary Materials to:

Graphene dots-ZnO Hybrid Nanostructures as High-Performance Chemiresistive Sensors for H_2S Detection

Federica Bucolo[a]*, Daniela Iannazzo,[a] Nesrine Hafiene,[a] Consuelo Celesti,[a] Roberto Di Pietro,[a] Ulderico Wanderlingh,[b,c] Sebastiano Vasi,[b,c] and Giovanni Neri[a]

[a] *Department of Engineering, University of Messina, 98166, Messina, Italy*

[b] *Department MIFT, University of Messina, 98166, Messina, Italy*

[c] *OPENFIS S.R.L. - Spin off accademico, University of Messina, Laboratorio A2AT3, Viale F. Stagno D'Alcontres 31, 98166 Messina, Italy*

*Corrispondence to: fbucolo@unime.it

CONTENT:

Figure S1. Tauc plot of ZnO NPs……………………………………………………………pag. S2

Figure S2. Tauc plot of GDs………………………….……………………………...pag. S2

Figure S3. Tauc plot of GDs-ZnO (1:1)…………………………….…………………..pag. S3

Figure S4. WH Plot of the ZnO peaks for the GDs-ZnO nanocomposite………………….pag. S3

Table S1. Data computed for the ZnO peaks by using the Williamson–Hall method……..pag. S4

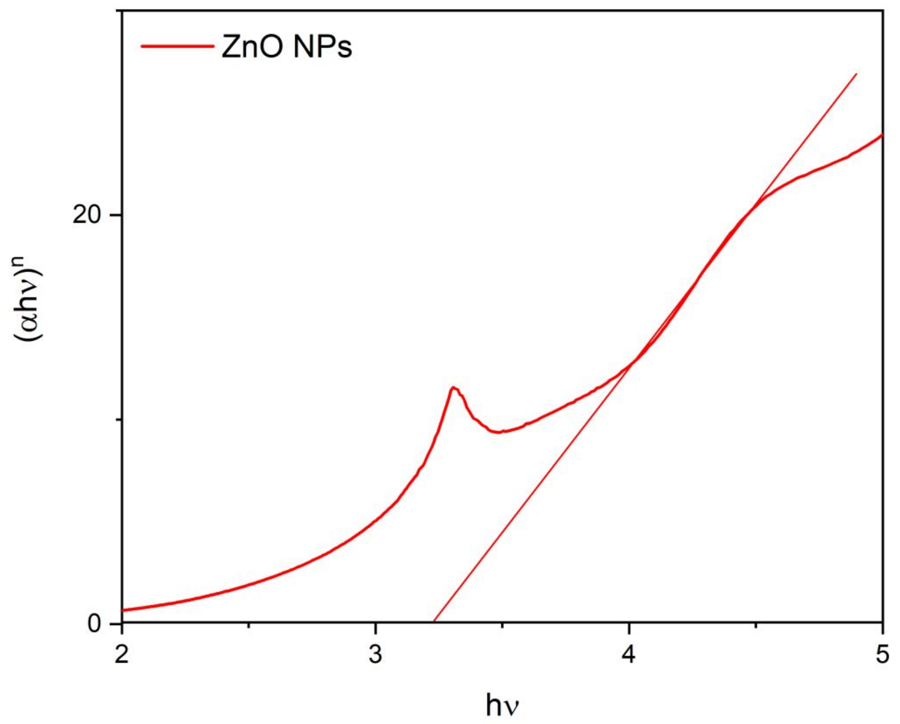

Figure S1. Tauc plot of ZnO NPs

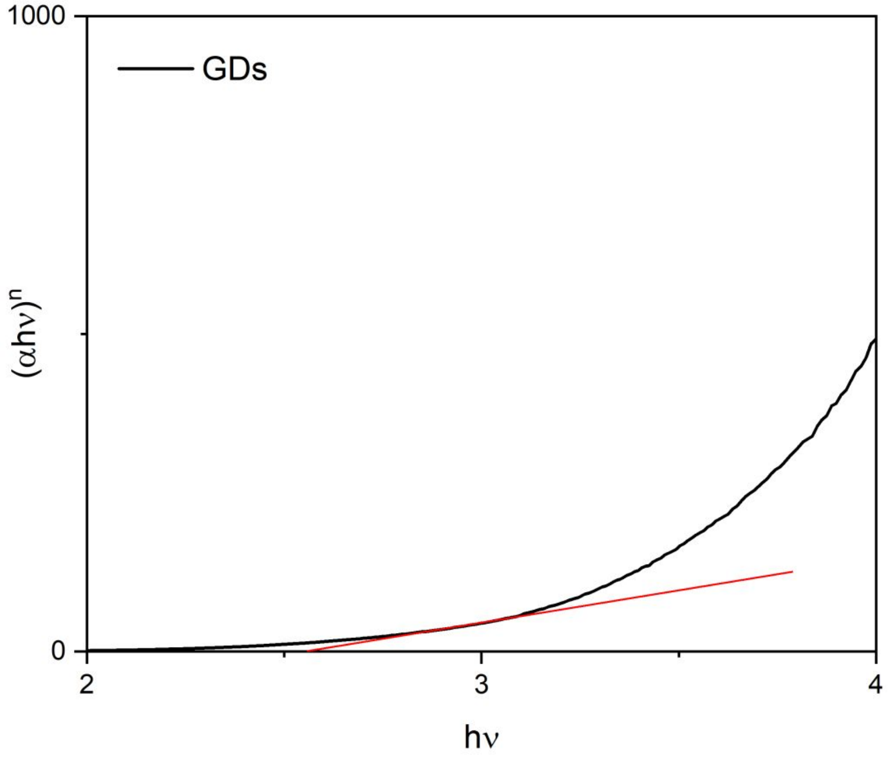

Figure S2. Tauc plot of GDs.

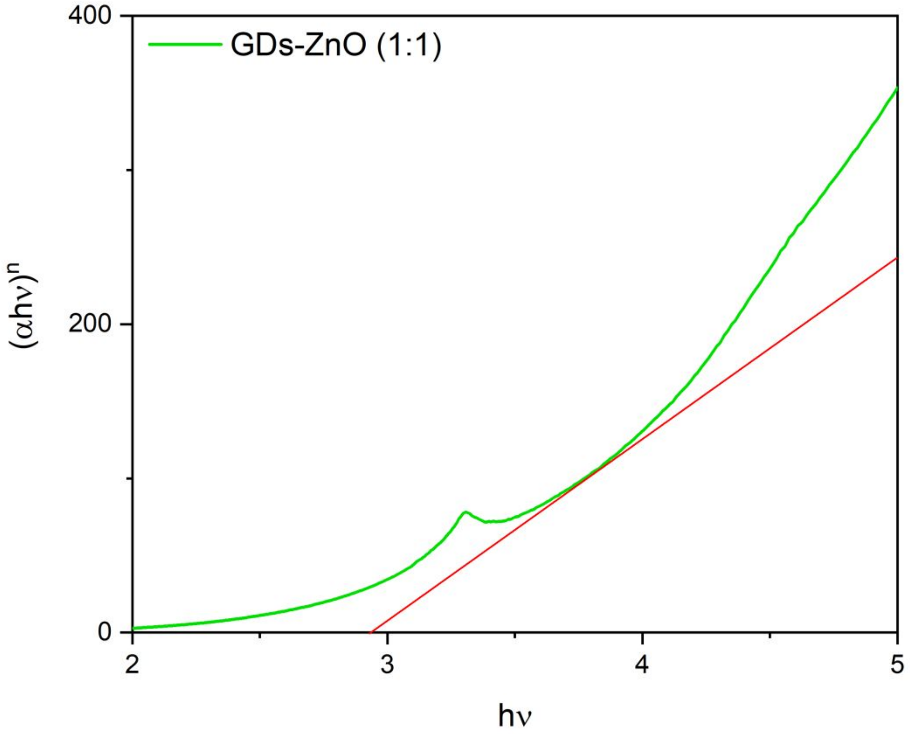

Figure S3. Tauc plot of GDs-ZnO (1:1)

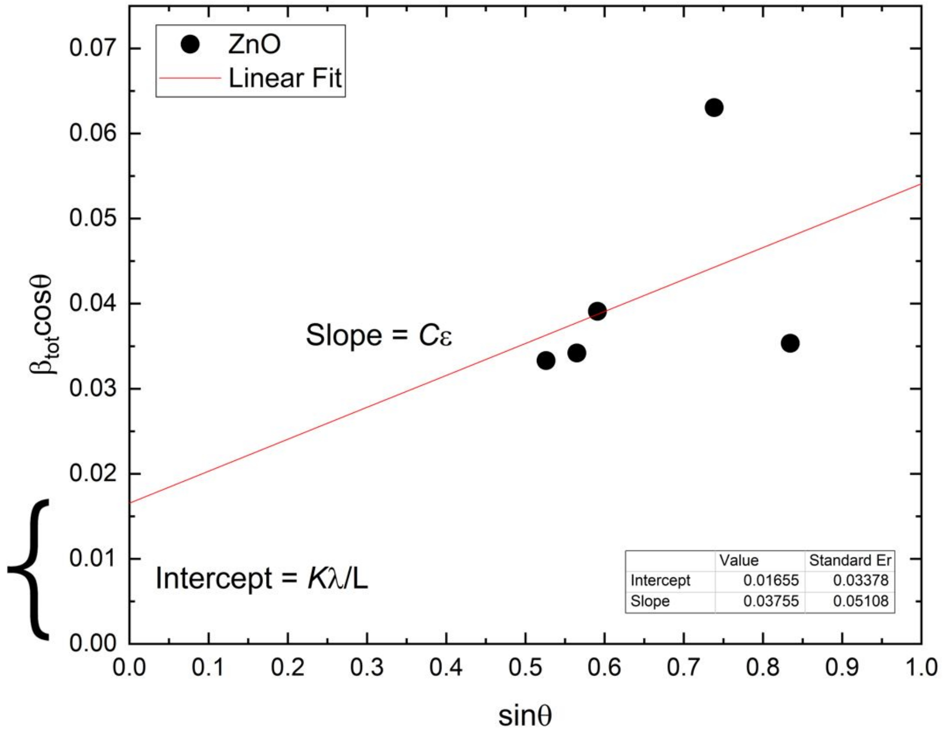

Figure S4. WH Plot of the ZnO peaks for the GDs-ZnO nanocomposite, together with the linear fit computed.

**hkl**	**θ**	**θ (rad)**	**cos θ**	**sin θ**	β_{tot}	β_{tot} **cos θ**
**100**	31.74	0.554	0.850	0.526	0.039	0.033
**002**	34.40	0.600	0.825	0.565	0.041	0.034
**101**	36.23	0.632	0.807	0.591	0.048	0.039
**102**	47.60	0.831	0.674	0.738	0.094	0.063
**110**	56.57	0.987	0.551	0.835	0.064	0.035

Table S1. Data computed for the ZnO peaks by using the Williamson–Hall method.
